# Prognostic factors and risk stratification in patients with castration-resistant prostate cancer receiving docetaxel-based chemotherapy

**DOI:** 10.1186/s12894-016-0133-y

**Published:** 2016-03-22

**Authors:** Shimpei Yamashita, Yasuo Kohjimoto, Takashi Iguchi, Hiroyuki Koike, Hiroki Kusumoto, Akinori Iba, Kazuro Kikkawa, Yoshiki Kodama, Nagahide Matsumura, Isao Hara

**Affiliations:** Department of Urology, Wakayama Medical University, 811-1 Kimiidera, Wakayama, 641-0012 Japan

**Keywords:** Castration-resistant prostate cancer, Docetaxel, Prognostic factor

## Abstract

**Background:**

While novel drugs have been developed, docetaxel remains one of the standard initial systemic therapies for castration-resistant prostate cancer (CRPC) patients. Despite the excellent anti-tumor effect of docetaxel, its severe adverse effects sometimes distress patients. Therefore, it would be very helpful to predict the efficacy of docetaxel before treatment. The aims of this study were to evaluate the potential value of patient characteristics in predicting overall survival (OS) and to develop a risk classification for CRPC patients treated with docetaxel-based chemotherapy.

**Methods:**

This study included 79 patients with CRPC treated with docetaxel. The variables, including patient characteristics at diagnosis and at the start of chemotherapy, were retrospectively collected. Prognostic factors predicting OS were analyzed using the Cox proportional hazard model. Risk stratification for overall survival was determined based on the results of multivariate analysis.

**Results:**

PSA response ≥50 % was observed in 55 (69.6 %) of all patients, and the median OS was 22.5 months. The multivariate analysis showed that age, serum PSA level at the start of chemotherapy, and Hb were independent prognostic factors for OS. In addition, ECOG performance status (PS) and the CRP-to-albumin ratio were not significant but were considered possible predictors for OS. Risk stratification according to the number of these risk factors could effectively stratify CRPC patients treated with docetaxel in terms of OS.

**Conclusions:**

Age, serum PSA level at the start of chemotherapy, and Hb were identified as independent prognostic factors of OS. ECOG PS and the CRP-to-albumin ratio were not significant, but were considered possible predictors for OS in Japanese CRPC patients treated with docetaxel. Risk stratification based on these factors could be helpful for estimating overall survival.

## Background

Prostate cancer is currently the most common malignancy in men from Western countries, and its occurrence has recently been increasing in Japan.

Because most prostate cancers grow in an androgen-dependent manner, androgen-deprivation therapy has been the initial treatment for recurrent or metastatic prostate cancer [1, 2]. However, under prolonged androgen deprivation, prostate cancer finally becomes refractory to hormonal manipulation and is then defined as castration-resistant prostate cancer (CRPC) [3, 4].

Recently, possible therapeutic strategies for CRPC have been increasing [5–8]. Novel therapies including enzalutamide, abiraterone acetate, cabazitaxel, sipuleucel-T, and radium 223 have been approved for therapy of CRPC patients. Enzalutamide and abiraterone acetate have shown their efficacy in not only the post-docetaxel setting but also the pre-docetaxel setting [9, 10]. In patients with no visceral metastasis, enzalutamide and abiraterone are recommended as well as docetaxel in the NCCN guideline. Moreover, in patients with visceral metastasis, these novel agents have been approved by FDA in the pre-chemotherapy setting. However, it was in 2014 that these agents were approved in Japan. In addition, the efficacy of novel therapies is still limited, and the prognoses of CRPC patients still remain poor.

To date, docetaxel, a natural taxane from *Taxus baccata*, has been established as effective and has been widely used in CRPC treatment [11, 12]. While novel drugs have been developed, docetaxel remains one of the standard initial systemic therapies for CRPC patients. In the EAU guideline 2014, docetaxel is still recommended as the first-line chemotherapeutic agent, especially in patients with evidence of progressive disease. Docetaxel is also recommended as the first-line drug in the NCCN guideline 2015. Despite of the excellent anti-tumor effect of docetaxel, its severe adverse effects, including myelosuppression, sometimes distress patients. Therefore, it would be helpful to predict the efficacy of docetaxel before treatment. Since novel agents such as enzalutamide and abiraterone acetate are now available, appropriate selection of CRPC patients using prognostic factors is crucial when choosing first-line therapy.

A predictive factor is a measurement that is associated with response or lack of response to a particular therapy. In contrast, a prognostic factor is a measurement that is associated with patient’s prognoses with or without treatment. Several prognostic factors in CRPC patients have been reported, and some nomograms or risk classifications have been developed. However, the magnitude of the benefit provided by each factor has varied among studies.

We previously reported that visceral metastases, including lung, liver and lymph nodes and excluding bone, and pretreatment anemia (hemoglobin < 11.3 g/dL) were two independent factors predicting overall survival in patients who received docetaxel chemotherapy for prostate cancer [13]. In a previous study, we collected the data from not only our hospital but also our related hospitals. Thus, the cohort in that study was rather heterogeneous regarding the indications, chemotherapy regimens, and amount of missing data. In the present study, patients only from our institute were targeted, and more variables, such as the neutrophil-to-lymphocyte ratio (NLR) and the C-reactive protein (CRP)-to-albumin ratio, were evaluated as predictors of overall survival.

The aims of this study were to evaluate the potential value of patient characteristics in predicting overall survival (OS) and to develop a risk classification for CRPC patients treated with docetaxel-based chemotherapy.

## Methods

### Patients and treatment

Patients with CRPC treated with docetaxel chemotherapy at the Wakayama Medical University Hospital (Wakayama, Japan) from June 2005 to May 2014 were included in this study. The eligibility criteria included histopathologically diagnosed adenocarcinoma of the prostate and confirmed failure of prior androgen deprivation therapy. If PSA increased after confirmation of the existence of antiandrogen withdrawal syndrome and alternative antiandrogen therapy, we judged that prior androgen deprivation therapy was a failure. No patient was administered abiraterone or enzalutamide or sipuleucel-T. In general, patients received 70 mg/m^2^ of docetaxel intravenously every 3 or 4 weeks. The recommended dose of docetaxel in the NCCN guidelines is 75 mg/m^2^, however the 70 mg/m^2^ dose is commonly used in Japan. This is because severe myelosuppression is more likely to develop in Japanese than Europeans and Americans. If necessary, dose reduction and interval extension were allowed, based on the patient’s overall condition. Prednisone 5 mg was routinely administered twice daily simultaneously with hormonal therapy for medical or surgical castration. Treatment with docetaxel was continued until disease progression, unacceptable adverse events, or the patient’s refusal. Disease progression was defined as increases in the number of evaluable lesions observed or in size of existing lesions by RECIST 1.1 on imaging tests and/or biological progression characterized by an elevated serum PSA level of 25 % and an absolute increase of ≥2 ng/mL than the nadir. As a general rule, PSA increase required at least two times.

This study was approved by the institutional review board of Wakayama Medical University (approval number 1672). Written informed consent to participate in this study was not obtained from patients since this study was a retrospective observational study for ordinary medical practice. Instead, information about this clinical study was disclosed at the web page of our hospital and posted at visitor consultation rooms in our hospital. If patients refused the use of their clinical data, we should have excluded their data from our study. However, no patient refused to provide his data for our study.

### Assessment

The variables, including patients’ characteristics at diagnosis (serum PSA level, Gleason score, and metastatic sites) and characteristics at the start of chemotherapy (age, ECOG performance status (PS), significant clinical pain, precedent treatment, serum PSA level, duration from initiation of androgen deprivation therapy, complete blood count, biochemical profile, combined drugs) were collected retrospectively. The NLR was calculated from the circulating neutrophil and lymphocyte counts. The CRP-to-albumin ratio was calculated from the CRP value and the albumin value using the formula: CRP value/albumin value) × 100.

The goal of the of the study was to determine the effect of patient characteristics on OS, calculated as the interval between the first day of docetaxel administration and the date of death or the last follow-up visit for censored (living) patients.

### Statistical analysis

All statistical analyses were performed using SPSS software. OS was determined according to the Kaplan-Meier method. Univariate and multivariate analyses of OS were performed to compare the prognostic factors in a Cox proportional hazards analysis. Continuous data were divided into 2 groups according to their median values. *P* < 0.05 was considered significant.

## Results

During the study period, 79 CRPC patients received docetaxel-based chemotherapy at our institution. The patients’ characteristics at diagnosis and at the start of chemotherapy are shown in Table 1. The median age was 72 years (range: 52–86 years). ECOG PS was <1 in 52 (65.8 %) patients and ≥1 in 27 (34.2 %) patients. Most patients had one or more metastases when they were diagnosed with prostate cancer. Overall, 9 (11.4 %) and 17 (21.5 %) patients underwent radical prostatectomy and radiation therapy, respectively. The median androgen deprivation therapy administration period was 31.4 months (range: 2.8–152.6 months). The median serum PSA level at chemotherapy initiation was 43.2 ng/mL (range: 2.7–3133.7 ng/mL). 54 patients (68.4 %) simultaneously received estramustine. Oral estramustine (560 mg) was administered on days 1 to 5 and days 8 to 12. However, adverse events associated with estramustine, such as thromboembolic events, did not develop.

**Table 1:** Patient characteristics

	*n* = 79
Median age (range), years	72 (52–86)
ECOG performance status, n (%)	
0	52 (65.8)
1	15 (19.0)
> = 2	12 (15.2)
Significant pain, n (%)	32 (40.5)
Median PSA at prostate cancer dianosis (range), ng/mL	125.2 (6.8–18778.0)
Gleason score, n (%)	
> =8	43 (54.4)
< =7	36 (45.6)
Metastatic site, n (%)	
bone	41 (51.9)
Lymph nodes	35 (44.3)
Lung	2 (2.5)
Liver	2 (2.5)
Prior treatment, n (%)	
Prostatectomy	9 (11.4)
Radiotherapy	17 (21.5)
Combined androgen blockade	79 (100)
Combination treatment, n (%)	
Bisphosphonate	23 (29.1)
Estramustine	54 (68.4)
Median PSA at docetaxel initiation (range), ng/mL	43.2 (2.7–3133.7)
Median androgen deprivation therapy administration period (range), months	31.4 (2.8–152.6)
Median serum markers at the start of docetaxel therapy (range)	
Hemoglobin, g/dL	11.9 (6.6–14.9)
NLR	2.9 (0.8–18.6)
Cre, mg/dL	0.8 (0.5–2.1)
ALP, IU/L	277 (1.9–4151.0)
LDH, IU/L	231 (123–594)
CRP-to-Albumin Ratio	7.3 (0.5–225.3)

*Abbreviations:* *ECOG* eastern cooperative oncology group, *PSA* prostate-specific antigen, *NLR* neutrophil-to-lymphocyte ratio, *Cre* creatinine, *ALP* alkaline phosphatase, *LDH* lactate dehydrogenase

The median number of chemotherapy cycles was 6 (range: 1–43). 20 patients (25.3 %) required dose reduction due to treatment intolerance or side effects. A total of 71 patients (89.9 %) discontinued docetaxel treatment because of disease progression (*N* = 39, 54.9 %), adverse events (*N* = 24, 33.8 %), patient’s refusal (*N* = 3, 4.2 %), and other reasons (*N* = 5, 7.0 %), respectively. The remaining 8 patients (10.1 %) were still undergoing docetaxel treatment during the course of the study. The median number of chemotherapy cycles in patients who discontinued treatment because of adverse events was 2.5 (range: 1–14).

**Fig. 1:**
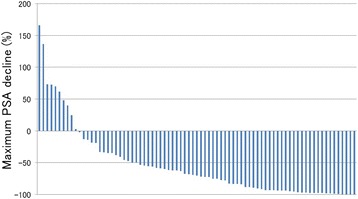
Waterfall plot of PSA response. PSA responses of ≥0 %, ≥30 %, and ≥50 % are seen in 69 (87.3 %), 64 (81.0 %), and 55 (69.6 %) patients, respectively

Waterfall plots of the PSA response are shown in Fig. 1. PSA responses ≥0 %, ≥30 %, and ≥50 % were observed in 69 (87.3 %), 64 (81.0 %), and 55 (69.6 %) of the total patients, respectively.

During the observation period (median 15.1 months, range: 1.8–53.4 months), 36 of all 79 patients (53.2 %) died of prostate cancer, and 6 (7.6 %) died of another cause. The median OS was 22.5 months, and the 1-year OS rate was 78.8 %.

Univariate and multivariate Cox proportional hazards regression models were used to investigate pre-treatment predictors of OS (Table 2). Among several predictors, age ≥ 72 years, ECOG PS ≥ 1, serum PSA level at the start of chemotherapy ≥ 40 ng/mL, duration from initiation of androgen deprivation therapy ≥ 30 months, Hb ≥ 12.0 g/dL, ALP ≥ 300 IU/L, and LDH ≥ 230 IU/L were identified as significant predictors for OS on univariate analysis. Furthermore, significant clinical pain, NLR ≥ 3, CRP-to-albumin ratio ≥ 7, and combination use of estramustine were not significant, but were considered possible predictors for OS. Of these factors, age ≥ 72 years and serum PSA level at the start of chemotherapy ≥ 40 ng/mL were independent unfavorable predictors of OS and Hb ≥ 12.0 g/dL was an independent favorable predictor of OS on multivariate analysis. ECOG PS ≥ 1 and CRP-to-albumin ratio ≥ 7 were not significant, but they were considered possible unfavorable predictors for OS.

**Table 2:** Univariate and multivariate analyses of associations between various pre-treatment parameters and overall survival

Variable	Univariate analysis			Multivariate analysis		
	HR	95 % CI	*P* value	HR	95 % CI	*P* value
Age > = 72 years	2.38	1.23–4.60	0.01	4.07	1.68–9.90	<0.01
ECOG PS > =1	2.92	1.59–5.51	<0.01	2.18	1.00–4.77	0.05
Significant pain	1.72	0.94–3.15	0.08	1.37	0.63–2.96	0.43
PSA at prostate cancer diagnosis > = 125 ng/mL	1.06	0.58–1.95	0.85			
Gleason score > = 8	0.7	0.38–5.78	0.26			
Metastatic site						
Bone	1.49	0.80–2.77	0.21			
Lymph node	1.05	0.57–1.93	0.89			
Prior treatment						
Prostatectomy	0.81	0.32–2.07	0.66			
Radiotherapy	1.14	0.57–2.27	0.72			
Combination therapy						
Bisphosphonate	1.02	0.52–1.99	0.96			
Estramustine	2.52	0.99–6.47	0.05	1.35	0.49–3.68	0.56
PSA at docetaxel initiation	3.45	1.76–6.77	<0.01	2.36	1.02–5.45	<0.05
Androgen deprivation therapy administration period > = 30 months	0.46	0.25–0.85	0.01	0.76	0.37–1.54	0.44
Serum markers at the start of docetaxel therapy						
Hemoglobin > = 12 g/dL	0.24	0.12–0.48	<0.01	0.24	0.10–0.59	<0.01
NLR > = 3	1.71	0.93–3.15	0.09	1.33	0.62–2.85	0.46
Cre > = 0.8 mg/dL	0.68	0.37–1.26	0.22			
ALP > =300 IU/L	3.27	1.70–6.30	<0.01	0.87	0.34–2.22	0.76
LDH > =230 IU/L	1.88	1.02–3.49	<0.05	1.24	0.62–2.46	0.55
CRP-to-Albumin Ratio > =7	1.7	0.92–3.12	0.09	2.34	0.91–6.05	0.08

*Abbreviations:* *ECOG* Eastern Cooperative Oncology Group, *PSA* prostate-specific antigen, *NLR* Neutrophil-to-Lymphocyte Ratio, *Cre* creatinine, *ALP* alkaline phosphatase, *LDH* lactate dehydrogenase

**Fig. 2:**
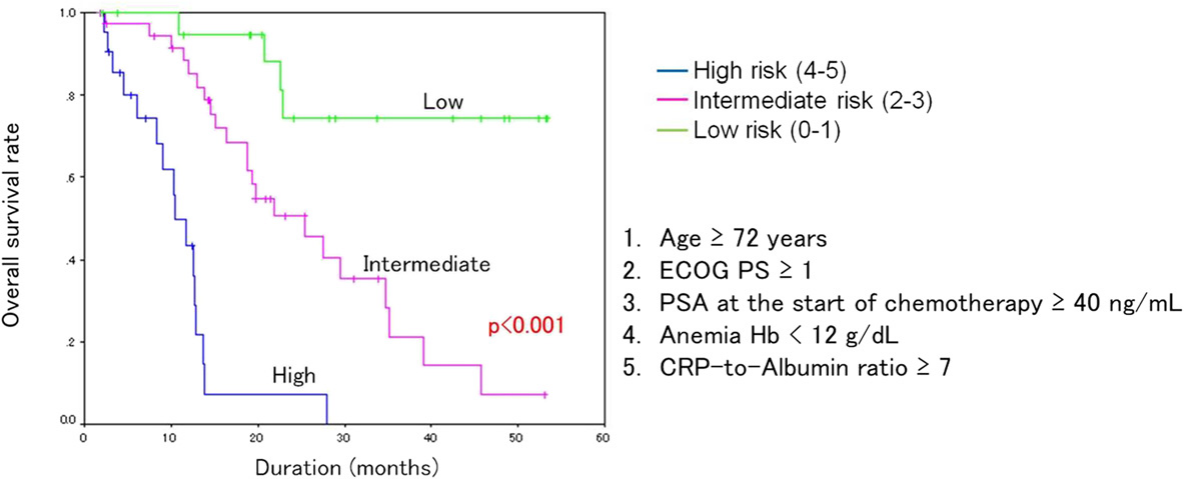
Kaplan-Meier curves for overall survival (OS) according to risk group classification

To develop a risk classification model to help in the appropriate selection of docetaxel chemotherapy in patients with CRPC, five pre-treatment factors, including advanced age (age ≥ 72 years), poor PS (ECOG PS ≥ 1), high serum PSA level at the start of chemotherapy (PSA ≥ 40 ng/mL), anemia (Hb < 12 g/dL), and high CRP-to-albumin ratio (CRP-to albumin ratio ≥ 7) were used, and the cohort was classified into three groups according to the presence of these five risk factors. Patients with 0–1, 2–3, and 4–5 risk factors were classified as low (*N* = 21), intermediate (*N* = 37), and high (*N* = 21) risk groups, respectively. This model effectively stratified patients in terms of OS according to risk group (*p* < 0.001), as shown in Fig. 2.

## Discussion

Two randomized trials demonstrated significant survival improvements in CRPC patients treated with docetaxel-based chemotherapy [11, 12], and it is widely used, including in Japan. Recently, several novel agents, including enzaltamide and abiraterone acetate, were shown to prolong overall survival in CRPC patients and have appeared on the market [5, 8]. However, docetaxel still remains a standard option, especially in CRPC patients with evidence of progressive disease. Accordingly, this provides clinicians with a wide choice between docetaxel and novel agents [14]. Therefore, it is crucial to identify prognostic factors and develop a risk stratification for CRPC patients treated with docetaxel.

Many prognostic models in patients with CRPC have been developed using pre and post chemotherapeutic factors. Although post chemotherapeutic factors, including PSA decline, tumor response, and pain response, are often used to evaluate patient’s prognosis [15, 16], it might be more beneficial for CRPC patients to predict the response and outcome before the initiation of chemotherapy. Armstrong et al. reported that four independent risk factors, including visceral metastases, bone scan progression, significant pain, and anemia (hemoglobin level < 13 g/dL), predicted OS well, and they developed a risk stratification model for CRPC patients treated with docetaxel [16]. This Armstrong risk classification (ARC) is highly reliable because it was developed from 656 CRPC patients treated with docetaxel and was also internally validated in 333 CRPC patients who were administered mitoxantrone from among the 1006 CRPC patients in the TAX 327 study [11]. However, few reports have externally validated the ARC in CRPC patients who were treated with docetaxel. Nakano et al. reported that the ARC could predict the prognosis in docetaxel-treated CRPC patients [17]. On the other hand, Shiota et al. reported that the ARC failed to stratify the cohort satisfactorily [18]. They showed that ALP value, visceral metastasis, and duration from initiation of hormone treatment were independent prognostic factors on multivariate analysis. Indeed, the reported prognostic factors for CRPC patients have differed widely among studies. The prognostic factors for CRPC patients reported in previous studies are shown in Table 3. Other than the above, Bamias et al. reported that baseline PSA >100, pain, weight loss, and simultaneous extraosseous and bone disease were associated with worse prognosis in CRPC patients [19]. Matsuyama et al. reported that a decrease of ≥ 50 % in the PSA, serum markers at the start of docetaxel therapy (PSA, ALP, and CRP), and the number of docetaxel courses were independent predictors of OS, and ALP, hemoglobin, and age at the start of docetaxel therapy were useful for deciding the duration of docetaxel therapy in CRPC patients [20].

**Table 3:** The prognostic factors for CRPC patients reported in previous studies

	Patient characteristics							Prognostic factor											
	Number of patients	PSA at DTX median	Age median	% bone meta	% visceral meta	% PSA >50 % reduction	OS median	Age	PS	Hb (Anemia)	CRP	PSA	PSA DT	PSA >50 % reduction	Pain	Bone scan progression	ALP	Visceral meta	Duration from initial ADT
Armstrong et al.	656	110	69	91	23	45–48	17.8–19.2	×	×	○		×	×		○	○		○	
Nakano et al.	78	19.7	70	54	5	23		×	×	×		×	○		○※1	○※1		○※1	×
Shiota et al.	97	81.3	70	83.5	15.5	44.3	20.8	×	×	×		×	×		×	×	○	○	○
Bamias et al.	94	84	71	87		54	16.2	×	×	×		○			○				×
Matsuyama et al.	279	35.2	71	60.5	7.9	57.5	26	×		×	○	○		○			○	×	
Ito et al.	80						14.5	×	×	○	○	×			×		×	×	
Nuhn et al.	238	91.2–112.9	68.3	90.3	16.5		14.4–18.3	×	×	○		○			×		×		
Present study	79	43.2	72	51.9	5	69.6	22.5	○	×	○	○※2	○			×		×		×

*Abbreviations:* *PSA* prostate-specific antigen, *DTX* docetaxel, *OS* overall survival, *PS* performance status, *Hb* haemoglibin, *CRP* C-reactive protein, *PSA DT* prostate-specific antigen doubling time, *ALP* alkaline phosphatase, *ADT* androgen deprivation therapy

○: Significant

×: Not significant

※1: The Armstrong risk classification was independent prognostic factor

※2: CRP-to-Albumin　Ratio was independent prognostic factor

The pre-chemotherapeutic prognostic factors are divided broadly into two categories. The first category reflects cancer progression. PSA value, PSADT, bone scan progression, and significant pain are included in this category. The second category represents the patient’s general condition. Age, ECOG PS, and CRP are included in the second category. The magnitude of the benefit provided by each factor has varied among studies.

In this retrospective study, prognostic factors were identified using only prechemotherapeutic factors in a single-institute Japanese cohort. The serum PSA level at the start of chemotherapy and anemia have been reported to be prognostic factors for CRPC patients in previous studies [16, 21, 22], and these factors were shown to be independent prognostic factors for CRPC patients treated with docetaxel in the present study. In addition, this study showed that age was also an independent prognostic factor and suggested that ECOG PS and the CRP-to-albumin ratio could be prognostic factors for CRPC patients. Accumulating evidence has indicated that cancer and inflammation are linked [23], and inflammation-based prognostic scores, including the NLR, Glasgow Prognostic Score (GPS), and Prognostic Nutritional Index (PNI), have been reported to have prognostic value in patients with various types of cancer in the last few years [24–28]. It has been reported that CRP and the NLR are useful clinical markers of the systemic inflammatory response in predicting OS in patients with CRPC treated with docetaxel [21, 22]. However, Kinoshita et al. reported that the CRP-to-albumin ratio might be an independent prognostic marker in patients with hepatocellular carcinoma and may have comparable prognostic ability to other established inflammation-based prognostic scores [29]. The present study suggested that this novel inflammation-based prognostic score could be a valuable prognostic marker in CRPC patients.

Our risk stratification could effectively stratify CRPC patients treated with docetaxel in terms of OS. Most of the prognostic factors included in the ARC, such as visceral metastases, bone scan progression, and significant pain, reflect the metastases of cancer. On the other hand, the prognostic factors included in the present risk stratification reflect the patient’s general condition. This difference was likely caused by the differences in the baseline characteristics of target patients. In the TAX 327 study, the median serum PSA level at chemotherapy initiation of the patients was over 100 ng/mL [11]. Bone metastases were observed in more than 90 % of patients, and visceral metastases were seen in about 20 %. However, in the present study, the median serum PSA level at chemotherapy initiation was 43.2 ng/mL, and the frequency of bone metastases or visceral metastases was less. Therefore, in the present study, a PSA response of ≥ 50 % from baseline was observed in about 70 % patients, and this PSA response rate was better than in previous clinical trials performed in Western countries, including the TAX327 trial [11, 12]. Moreover, the median OS duration in this cohort was 22.5 months, while the median OS duration in the TAX327 cohort was <20 months. The difference of disease burden between our cohort and the TAX327 cohort caused these differences of PSA response and OS. Considering these facts, it appears that factors representing disease progression might be associated with OS in patients with progressive disease, while factors reflecting the patient’s general condition might be correlated with OS in patients with less progressive disease – namely, prostate cancer with low serum PSA level at chemotherapy initiation or without bone metastases and visceral metastases. Lower disease burden in our cohort might be one of the reasons that significant pain, included in ARC, was not a significant factor in this study.

With the advent of novel agents, the demand for docetaxel in CRPC patients might slightly decrease. However, the results of the CHAARTED trial indicate the new effect of docetaxel for prostate cancer, and the importance of docetaxel will never be lost. Our study focused on the prognostic factors in CRPC patients receiving docetaxel-based chemotherapy, but the study about the prognostic factors in hormone sensitive prostate cancer (HSPC) patients receiving docetaxel-based chemotherapy will be needed.

This study had some limitations. First, the sample size was small, and the observation period might not have been long enough to determine the actual OS. Second, this study was a retrospective study using data from a single institution. Thirdly, many patients in our cohort received estramustine. It is less likely to combine estramustine to docetaxel based chemotherapy in Europe and United States. In fact, EAU and NCCN guidelines do not recommend to combine estramustine with docetaxel based chemotherapy. However, serious adverse events of estrogen, such as embolism, are reported to be rather rare in Japanese patients [30]. Finally, most patients required docetaxel dose reductions because of adverse events. Additional larger confirmatory studies are warranted to validate our results.

## Conclusions

Age, serum PSA level at the start of chemotherapy, and Hb were identified as independent prognostic factors of OS, and ECOG PS and the CRP-to-albumin ratio were not significant but were considered as possible predictors for OS in Japanese CRPC patients treated with docetaxel. Risk stratification based on these factors could be helpful for estimating OS.

## Abbreviations

ALP: alkaline phosphatase
ARC: Armstrong risk classification
CRP: C-reactive protein
CRPC: castration-resistant prostate cancer
EAU: European Association of Urology
ECOG: Eastern Cooperative Oncology Group
GPS: Glasgow Prognostic Score
Hb: hemoglobin
LDH: lactate dehydrogenase
NCCN: National Comprehensive Cancer Network
NLR: neutrophil-to-lymphocyte ratio
OS: overall survival
PNI: Prognostic Nutritional Index
PS: performance status
PSA: prostate specific antigen
PSADT: PSA doubling time
